# Severe Guillain-Barré syndrome associated with chronic active hepatitis C and mixed cryoglobulinemia: a case report

**DOI:** 10.1186/s12879-019-4278-7

**Published:** 2019-07-17

**Authors:** Alexandre Chlilek, Claire Roger, Laurent Muller, Marie-Josée Carles, Robin Stephan, Didier Laureillard, Jean-Philippe Lavigne, Jean-Yves Lefrant, Albert Sotto

**Affiliations:** 1 Service de Microbiologie, CHU Carémeau, 30029 Nîmes, France; 2 Service de Réanimation Chirurgicale, CHU Carémeau, 30029 Nîmes, France; 3 Service de Maladies Infectieuses et Tropicales, CHU Carémeau, 30029 Nîmes, France; 4 Université de Montpellier, 186 Chemin du Carreau de Lanes, 30908, cedex 02 Nîmes, France

**Keywords:** Chronic hepatitis C, Hepatitis C virus, Guillain-Barre syndrome, Mixed cryoglobulinemia, Extra-hepatic manifestations

## Abstract

**Background:**

We describe a case of severe Guillain-Barre syndrome (GBS) associated with chronic active hepatitis C and mixed cryoglobulinemia (MC). To our knowledge, this association between GBS and hepatitis C virus (HCV) infection has been rarely reported.

**Case presentation:**

A 56-year-old man developed symmetrical muscle weakness in all extremities, areflexia and sensorial disorder followed by acute respiratory failure associated with chronic active hepatitis C, which was confirmed by the presence of anti-HCV antibodies in the serum and persistence of HCV RNA viral load for more than 6 months. Chronic hepatitis C was further complicated by type 3 MC. Electromyography showed peripheral nerve injury (mainly in axon). A severe acute motor sensory axonal neuropathy (AMSAN) was diagnosed. After treatment with intravenous immunoglobulin and plasma exchange followed by antiviral therapy by direct-acting antiviral agent, patient showed progressive recovery and was transferred 3 months after his first admission to a rehabilitation center.

**Conclusions:**

Our case reported a severe GBS associated with HCV infection and MC. EMG classified for the first time the subtype of GBS (severe AMSAN) correlated with severe clinical form. HCV infection should be screened in high-risk patients to prevent silent progression of the chronic hepatitis C and its potentially severe extra-hepatic manifestations.

## Background

Hepatitis C virus (HCV) infection, the second most common chronic viral infection in the world, is an important public-health concern and leads to substantial mortality [1]. HCV causes asymptomatic acute hepatitis in 80 to 90% of cases and chronic hepatitis in 55 to 85% of infected individuals in the absence of treatment [2]. Patients with chronic active hepatitis C are known to be at risk of developing liver complications, i.e., cirrhosis and liver cancer but they are also susceptible to present numerous extra-hepatic manifestations (EHMs) which are often underestimated [3]. Mixed cryoglobulinemia (MC) syndrome, a systemic autoimmune vasculitis, is the most prevalent EHM but others have been recently reported including many neurological manifestations [3–6].

Guillain-Barre syndrome (GBS) is the most frequent and severe acute paralytic neuropathy worldwide [7]. Acute respiratory failure can complicate GBS in 20–30% of cases and requires admission in intensive care unit (ICU) [7]. The mechanism of GBS is based on demyelinating lesions of peripheric nerves which often occur after infectious episode by autoimmune cross reaction [8]. Over the past decades, the cases of GBS caused by *Campylobacter jejuni* and numerous viral agents have greatly increased [7]. Recently, the emerging relation between GBS and Zika virus infection has aroused general interest as the global epidemic spreads [9]. To our best knowledge, only seven cases of GBS associated with HCV infection have been reported so far [10–13]. Here, we report one case of severe GBS associated with chronic active hepatitis C and MC.

## Case presentation

**Fig. 1:**
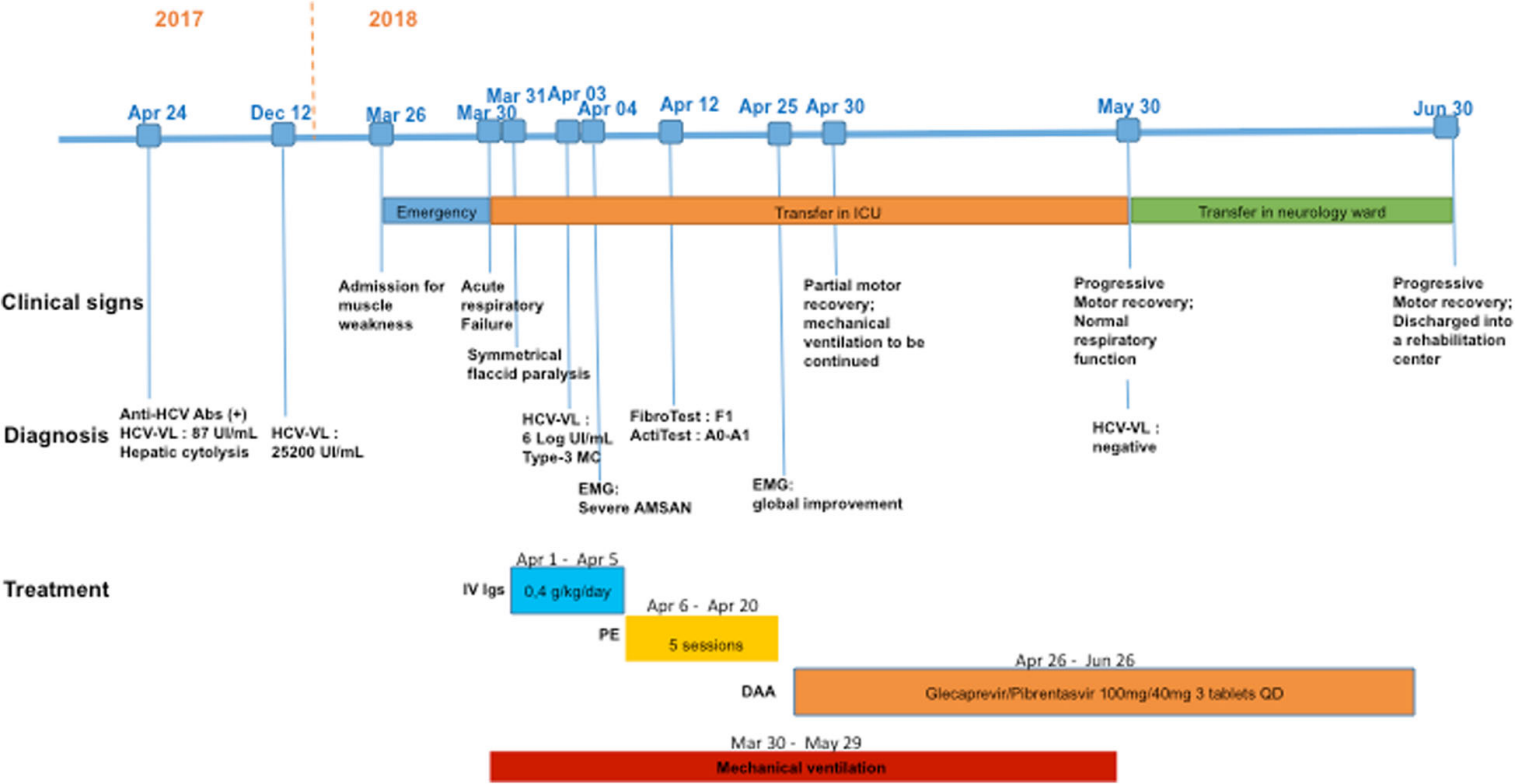
Medical history and treatment of a severe Guillain-Barre syndrome (*GBS*) associated with chronic active hepatitis C with mixed cryoglobulinemia. *Abs* Antibodies; *VL* Viral Load; *ICU* Intensive Care Unit, I*V Igs* Intravenous immunoglobulins, *DAA* Direct-acting antiviral, *QD* Once a day

A 56 year-old male patient was admitted to our hospital emergency for muscle weakness of all limbs and areflexia (Fig. 1). His past medical history was marked by intravenous drug addiction (started at the age of 28 and currently treated by buprenorphine) and chronic respiratory failure after chronic obstructive pulmonary disease (tobacco consumption estimated at 36 pack-years). A serum test performed 1 year ago showed positive anti-HCV antibodies on ELISA (Enzyme Linked ImmunoSorbent Assay) confirmed by LIA (Line ImmunoAssay) with HCV RNA viral load at 87 IU/mL. At the same time, liver function tests indicated elevated levels of alanine aminotransferase (ALT) to 256 IU/L (10–50 IU/L) and aspartate aminotransferase (AST) to 123 IU/L (10–50 IU/L). Chronic HCV infection was proven by persistence of viral load with a 3 log increase (25 200 U/mL) on a blood test withdrawn 4 months ago.

On admission, neurological physiological examination revealed consciousness, quadriplegia of all the limbs with emphasis in arms and right side, sensorial disorders in legs, decreased muscle tension and tendon areflexia in all the limbs, without aphasia, facial nerve palsy or diplopia. Four days after emergency admission, the patient was transferred to our ICU for acute respiratory failure. Physical examination showed dyspnea, tachypnea and sharp pulling necessitating rapid sequence intubation and mechanical invasive ventilation associated with loss of consciousness (Glasgow Coma Score of 10). There was no jaundice or abdominal pain. Blood gas demonstrated severe hypoxemia (pO_2_ 43.8 mmHg) and hypercapnia associated with respiratory acidosis (pCO_2_ 99.1 mmHg, pH 7.06, HCO_3_^−^ 28.2 mmol/L). Blood tests showed lactates 3.0 mmol/L, white blood cells 31 × 10^9^/L, Hemoglobin 12.6 g/dL, C-reactive protein 14.9 mg/L. The liver assessment was normal. Cardiac ultrasound exam ruled out any cardiac origin of acute respiratory failure. Chest X ray did not show any sign of pneumonia but *Haemophilus influenzae* was identified in pulmonary samples. Cerebrospinal fluid (CSF) examination showed an absence of leukocyte, 5.5 mmol/L glucose level, and 33 mg/dL protein level, which didn’t suggest albuminocytologic dissociation. Cerebral and medullary magnetic resonance imaging indicated no abnormalities.

Based on initial neurological examination performed in the Department of emergency and respiratory involvement in the ICU, the physicians suspected that the patient presented with GBS. On day 2 after admission in ICU, treatment with intravenous immunoglobulins at a dose of 0.4 g/kg per day for 5 days was initiated. Afterwards, five plasma exchange sessions were implemented over 2 weeks.

On day 4 after admission in the ICU, serological study for anti-HCV antibodies was positive and HCV RNA blood viral load was raised to 1 710 000 IU/mL with HCV assignment into genotype 4. Liver function tests indicated new increased levels of ALT to 103 IU/L (10–50 IU/L) and AST to 86 IU/L (10–50 IU/L). No serological evidence was found for *Campylobacter jejuni* infection, human immunodeficiency virus, hepatitis B virus and syphilis. Hepatitis A virus and cytomegalovirus serology indicated positive IgG. Serum electrophoresis showed marked polyclonal hypergammaglobulinemia with elevated gammaglobulin level to 20.9 g/L (8.0–13.5 g/L) including 17.5 g/L IgG (6.7–12.4 g/L) and 2.0 g/L IgM (0.6–1.6 g/L). The screening for cryoglobulin by serum cryoprecipitation was positive and immunoelectrophoresis indicated the presence of polyclonal IgG combined with polyclonal IgM. These assays were confirmed twice ten days apart, attesting type-3 MC. In addition, one other immunological test reported decreased serum complement level to 39 U/mL (42–95 U/mL) with reduction in C4 complement fraction to 0.14 g/L (0.18–0.42 g/L).

Electromyography (EMG) carried out at day 5 after admission in ICU showed prolonged distal motor latency, major decrease of motor and sensory nerve action potentials amplitude, absence of voluntary activity and several spontaneous electromyographic activity in all the territories studied, demonstrating evidence of severe acute motor sensory axonal neuropathy (AMSAN) with demyelinating lesions compatible with a peculiar type of GBS.

FibroTest and ActiTest performed on day 14 after admission in ICU showed minimal fibrosis stage (F1) and minimal activity grade (A0-A1), respectively. After plasma exchange sessions, the patient was treated for 2 months with direct-acting antiviral agent (DAA) (glecaprevir/pibrentasvir).

During the first month, the patient showed very slow motor recovery and difficulties with weaning from mechanical ventilation requiring performing tracheostomy. Control EMG performed on day 27 indicated marked improvements in distal motor latencies and motor and sensory nerve action potentials amplitude, major decrease of spontaneous electromyographic activities and recovery of voluntary activity in all the territories studies, suggestive of GBS being recovered.

Afterwards, the patient improved with progressive recovery of muscle strength and normal respiratory function. After one month of DAA therapy, HCV ARN was undetectable and liver enzymes were normal. There was no relapse. The patient was transferred 3 months after his first admission to a rehabilitation centre.

## Discussion and conclusion

GBS is an acute immune-mediated polyradiculoneuropathy that results in rapidly progressing symmetric motor paralysis and decreased tendon reflexes or areflexia, often with sensory deficits and cranial nerve involvement. The acute progression of limb weakness, 1–2 weeks after immune stimulation often triggered by an infectious disease, proceeds to its peak clinical deficit in 2–4 weeks [14]. Examination of the CSF classically shows cytoalbuminological dissociation, i.e., the combination of a normal cell count and increased protein level. GBS is also a clinically diverse disorder with several distinctive variants and atypical cases. Nerve conduction studies can help clinicians to make the diagnosis and to consider prognosis enabling them to divide GBS into acute inflammatory demyelinating polyneuropathy, acute motor axonal neuropathy, or AMSAN [15]. To treat GBS, IVIgs or plasma exchange have proved to be effective and should be started as soon as possible especially in patients with rapidly progressive weakness before irreversible nerve damage taken place [16]. In our case, GBS was suspected once the patient was found as having acute respiratory failure after symmetrical paralysis of all limbs,. We didn’t get the proof from CSF because of the absence of albuminocytologic dissociation. In fact, normal protein level is reported in approximately 50% of cases when determined in the first 4 days after the onset of the disease [14] which does not make the diagnosis unlikely or even exclude GBS. EMG confirmed the diagnosis and demonstrated AMSAN. This electrophysiological GBS subtype is very scarce in Europe and North America [17]. It is generally correlated to more severe limb weakness, poorer outcome than the others subtypes and the patients often require mechanical ventilation [18]. However, most of AMSAN patients have past history of diarrhea or serological evidence of *C. jejuni* infection [18], which was not highlighted here.

The patient had chronic active hepatitis C. In developed countries, HCV infections are mainly caused in high risk populations, especially persons who inject drugs [19]. In the absence of previous HCV serology, it is not excluded that the patient had already chronic HCV infection on the blood test withdrawn one year before admission in our ICU, suggestive of subclinical transition from a clinically silent acute HCV infection. HCV RNA viral load was positive on the first blood test and persistent more than 6 months before the onset of neurological symptoms. Meanwhile, liver enzymes were initially positive on the first blood test then negative on the liver function test withdrawn at emergency admission with a new increase 4 days after in the ICU. This biological profile is compatible with chronic hepatitis C where transaminases are known to fluctuate rapidly. Moreover, cases of asymptomatic rise of transaminases contemporaneous of the acute phase of GBS have already been described [10, 12]. Thus, we could hypothesize that chronic hepatitis C may have been the trigger of GBS.

Moreover, the patient presented type-3 MC. MC is based on the presence of mixed cryoglobulins in the circulation consisting either of a monoclonal Ig, plus a polyclonal (type II cryoglobulins), or two polyclonal Igs (type III). Serum cryoglobulins are frequently detected in HCV-infected patients, especially in Southern Europe (50–70%) [20]. MC syndrome is generally classified as a systemic small-vessel vasculitis that affects up to 10–15% of subjects chronically infected with HCV [20] and may involve kidneys, joints, skin and peripheral nerves. Peripheral neuropathy, the most common neurologic complication of HCV [4], is more prominent with HCV associated MC where the most frequently described form is a symmetrical sensory-motor polyneuropathy with predominant sensory features [21]. However, the presence of MC doesn’t increase overall risk of developing a neuropathy [22] and the pathophysiology of neuropathy associated with HCV is not definitively known [21]. Furthermore, the patient recovered without the use of rituximab. Immunosuppressive agents as rituximab constitute the best non-antiviral therapeutic option for refractory or severe forms of MC associated with chronic HCV infection [23]. However, although the clinical experience on the use of the new DAAs currently remains very limited, recent evidence-based recommendations advise the use of these new antiviral treatments for all patients presenting HCV-related MC with no clear indications on how to combine them and non-antiviral treatment [24].

As far as we know, only seven cases of GBS associated with hepatitis C have been reported. In 1993, De Klippel et al. reported the first case in Belgium [10]. A woman manifested distal paresis of both legs and arms with areflexia and paresthesia. Anti-HCV antibodies were detectable in the serum. Nerve conduction studies met the criteria of GBS and were consistent with a demyelinating neuropathy. Blood test results, rapid normalization of liver enzymes and complete absence of fibrosis on liver biopsy suggested to clinicians GBS associated with resolving acute HCV infection. In 1995, a second case of HCV infection associated with GBS was described in France in a patient presenting chronic active hepatitis C [11]. In 1998, Lacaille et al. reported two others cases [12]. The third case was a 51-year-old man who presented rapidly progressive paresis of four limbs, areflexia and paresthesia further complicated by paralysis of respiratory muscles involving mechanical ventilation. Cerebrospinal fluid was normal and nerve conduction studies showed an asymmetrical axono-myelinic polyradiculoneuritis suggestive of GBS. Anti-HCV antibodies were positive and liver biopsy indicated chronic active hepatitis. The fourth case was a young woman who developed paresis and sensory loss of all her four limbs with areflexia two years after the end of α-interferon treatment for chronic hepatitis C with moderate activity. CSF examination showed slightly elevation of proteins and normal cell count. EMG showed partial peripheral neurogenic symptoms, with normal conduction speed. After IVIg therapy, the patient gradually recovered but experienced a relapse. Finally, she fully improved after four courses of plasma exchange. In 2001, Massengo et al. reported the fifth case presenting GBS caused by hepatitis C associated with cryoglobulinemia [13]. In 2008 and 2010, two others cases with respectively atypical and typical features for GBS associated with pegylated interferon alpha-2a have been published [25, 26]. It was worth to mention that GBS has also been reported in association with non-A, non-B hepatitis [27–29]; perhaps these cases were due to HCV infection.

There were some limitations in our case. Firstly, we didn’t get another CSF sample to prove albuminocytologic dissociation. However, according to the recently proposed new diagnostic classification, GBS could be diagnosed by clinical characteristics and electrophysiological examinations, without the need of CSF examinations, and whether or not they disclose existing diagnostic criteria [30]. On the other hand, we had not previous serum tests to explore history of HCV infection specially to date the transition from acute to chronic infection.

In conclusion, we describe a case of GBS associated with chronic active hepatitis C and MC. GBS should be added to the list of EHMs of hepatitis C as well as HCV as an additional cause of GBS. Moreover, we classified the subtype of GBS (severe AMSAN) based on the electrophysiology characteristics and correlated to the clinical presentation. We thus observed that chronic active hepatitis C is a general disorder which can lead to severe peripheral neuropathy requiring admission in ICU in absence of treatment. Hence the need, in the future, of setting up effective programs to make HCV screening and treatment easier in high-risk populations who have chronic hepatitis C.

## Data Availability

The datasets analysed during the current case report are available from the corresponding author on reasonable request.

## Abbreviations

ALT: Alanine aminotransferase
AMSAN: Acute motor sensory axonal neuropathy
AST: Aspartate aminotransferase
CSF: Cerebrospinal fluid
DAA: Direct-acting antiviral agent
EHM: Extra-hepatic manifestation
ELISA: Enzyme linked immunosorbent assay
EMG: Electromyography
GBS: Guillain-Barre syndrome
HCV: Hepatitis C virus
LIA: Line immunoassay
MC: Mixed cryoglobulinemia
